# Complete Loss of EPCAM Immunoexpression Identifies *EPCAM* Deletion Carriers in MSH2-Negative Colorectal Neoplasia

**DOI:** 10.3390/cancers12102803

**Published:** 2020-09-29

**Authors:** Míriam Cuatrecasas, Iñigo Gorostiaga, Cristina Riera, Esteban Saperas, Gemma Llort, Irmgard Costa, Xavier Matias-Guiu, Cristina Carrato, Matilde Navarro, Marta Pineda, Núria Dueñas, Joan Brunet, Vicente Marco, Isabel Trias, José Ignacio Busteros, Gemma Mateu, Francesc Balaguer, María-Teresa Fernández-Figueras, Manel Esteller, Eva Musulén

**Affiliations:** 1 Department of Pathology, Center of Biomedical Diagnosis (CDB), Hospital Clínic, 08036 Barcelona, Spain; mcuatrec@clinic.cat; 2 Universitat de Barcelona (UB), 08007 Barcelona, Spain; xmatias@bellvitgehospital.cat (X.M.-G.); mesteller@carrerasresearch.org (M.E.); 3 Centro de Investigación Biomédica en Red de Enfermedades Hepáticas y Digestivas (CIBERehd), 08036 Barcelona, Spain; fprunes@clinic.cat; 4 Department of Pathology, Hospital Universitario de Araba, 01009 Vitoria-Gasteiz, Spain; igorostiagaltuna@gmail.com; 5 Gastroenterology Department, Hospital Universitari General de Catalunya-Grupo Quirónsalud, Sant Cugat del Valles, 08195 Barcelona, Spain; cristina.riera@quironsalud.es (C.R.); esteban.saperas@quironsalud.es (E.S.); 6 Universitat Internacional de Catalunya (UIC), Sant Cugat del Vallès, 08017 Barcelona, Spain; maite.ffigueras@quironsalud.es; 7 Oncology Department, Parc Taulí Hospital Universitari, Sabadell, 08208 Barcelona, Spain; gllort@tauli.cat; 8 Oncology Department, Consorci Sanitari de Terrassa, Terrassa, 08208 Barcelona, Spain; 9 Department of Pathology, Parc Taulí Hospital Universitari, Sabadell, 08208 Barcelona, Spain; icosta@tauli.cat; 10 Department of Pathology, Hospital Universitari de Bellvitge, Institut d’Investigació Biomèdica de Bellvitge (IDIBELL), L’Hospitalet de Llobregat, 08908 Barcelona, Spain; 11 Department of Pathology, Hospital Universitari Arnau de Vilanova, 25198 Lleida, Spain; 12 Universitat de Lleida, IRBLLEIDA, 25003 Lleida, Catalonia, Spain; 13 Centro de Investigación Biomédica en Red Cancer (CIBERONC), 28029 Madrid, Spain; mnavarrogarcia@iconcologia.net (M.N.); mpineda@iconcologia.net (M.P.); nduenas@iconcologia.net (N.D.); jbrunet@iconcologia.net (J.B.); 14 Department of Pathology, Hospital Universitari Germans Trias i Pujol, 08916 Badalona, Barcelona, Spain; ccarrato.germanstrias@gencat.cat; 15 Hereditary Cancer Program, Catalan Institute of Oncology, Institut d’Investigació Biomèdica de Bellvitge (IDIBELL), ONCOBELL Program, Hospitalet de Llobregat, 08908 Barcelona, Spain; 16 Hereditary Cancer Program, Catalan Institute of Oncology, Institut d’Investigació Biomèdica de Girona (IDIBGI), Universitat de Girona, 17190 Girona, Spain; 17 Department of Pathology, Hospital Quirónsalud Barcelona, 08023 Barcelona, Spain; vicente.marco@quironsalud.es; 18 Department of Pathology, Hospital Platón, 08006 Barcelona, Spain; isabel.trias@hospitalplato.com; 19 Department of Pathology, Hospital Universitario Príncipe de Asturias, 28805 Alcalá de Henares, Madrid, Spain; joseignacio.busteros@salud.madrid.org; 20 Department of Pathology, University Hospital Josep Trueta, 17007 Girona, Spain; gmateu.girona.ics@gencat.cat; 21 Gastroenterology Department, Institut de Malalties Digestives i Metabòliques, Hospital Clínic, 08036 Barcelona, Spain; 22 Department of Pathology, Hospital Universitari General de Catalunya-Grupo Quirónsalud, Sant Cugat del Vallès, 08190 Barcelona, Spain; 23 Josep Carreras Leukaemia Research Institute (IJC), 08916 Badalona, Barcelona, Spain; 24 Institució Catalana de Recerca i Estudis Avançats (ICREA), 08010 Barcelona, Spain

**Keywords:** lynch syndrome, *EPCAM*, immunohistochemistry, colorectal cancer, colon polyps

## Abstract

**Simple Summary:**

Colorectal carcinomas from patients with Lynch syndrome (LS) due to *EPCAM* deletions show loss of MSH2 expression. The aim of our study was to evaluate the usefulness of EPCAM expression in identifying carriers of *EPCAM* deletion among patients with MSH2-negative lesions. MSH2 and EPCAM immunohistochemistry was performed in a large series of lesions (190) composed of malignant and benign neoplasms as well as precursor lesions of different organs from 71 patients with suspected LS due to *MSH2* alterations. Germ-line analysis confirmed LS in 68 patients due to *MSH2* mutations (53) and *EPCAM* deletions (15). Among colorectal lesions with lack of MSH2 expression, only 17 were EPCAM-negative and belonged to patients with *EPCAM* deletions. We confirm that loss of EPCAM expression identifies *EPCAM* deletion carriers with 100% specificity and we recommend adding EPCAM IHC to the algorithm of MSH2-negative colorectal neoplasia.

**Abstract:**

The use of epithelial cell adhesion molecule (EPCAM) immunohistochemistry (IHC) is not included in the colorectal cancer (CRC) screening algorithm to detect Lynch syndrome (LS) patients. The aim of the present study was to demonstrate that EPCAM IHC is a useful tool to guide the LS germ-line analysis when a loss of MSH2 expression was present. We retrospectively studied MSH2 and EPCAM IHC in a large series of 190 lesions composed of malignant neoplasms (102), precursor lesions of gastrointestinal (71) and extra-gastrointestinal origin (9), and benign neoplasms (8) from different organs of 71 patients suspicious of being LS due to *MSH2* alterations. LS was confirmed in 68 patients, 53 with *MSH2* mutations and 15 with *EPCAM* 3′-end deletions. Tissue microarrays were constructed with human normal tissues and their malignant counterparts to assist in the evaluation of EPCAM staining. Among 154 MSH2-negative lesions, 17 were EPCAM-negative, including 10 CRC and 7 colorectal polyps, and 5 of them showed only isolated negative glands. All lesions showing a lack of EPCAM expression belonged to patients with *EPCAM* 3′-end deletions. EPCAM IHC is a useful screening tool, with 100% specificity to identify LS patients due to *EPCAM* 3′-end deletions in MSH2-negative CRC and MSH2-negative colorectal polyps.

## 1. Introduction

Lynch syndrome (LS) is an inherited cancer predisposition syndrome caused by the alteration of mismatch repair (MMR) system genes [1,2]. Colorectal cancer (CRC) is the most frequent neoplasm in these patients, although the tumor spectrum varies according to the affected gene. Currently, universal screening is recommended in all new diagnosed CRC to rule out LS, mostly based on the immunohistochemical (IHC) expression of MMR gene proteins but, also, by PCR detection of microsatellite instability (MSI) [3]. Only 2% to 3% of all LS cases are due to deletions of the 3′-end of the epithelial cell adhesion molecule (*EPCAM*) gene and present almost exclusively with gastrointestinal (GI) tumors, predominantly CRC [4,5,6,7]. The *EPCAM* gene, located at chromosome 2q, consists of nine exons and is placed just before the *MSH2* gene. The lack of the 3′-end of *EPCAM* produces hypermethylation of the contiguous *MSH2* gene promoter, which is silenced [8,9]. In this situation, a concomitant lack of both MSH2 and EPCAM protein expressions occur in CRC, which specifically identifies *EPCAM* 3′-end deletion carriers [10,11,12,13,14,15]. In some cases, the *EPCAM* 3′-end deletion may extend to the first *MSH2* exons of the 5′ end, including the promotor region, with no *MSH2* hypermethylation. This double-*EPCAM-MSH2* deletion occurs with extra-GI neoplasms, mostly endometrial carcinoma [16,17]. Since the LS tumor spectrum is variable according to the involved gene, it is crucial to know the specific germ-line alteration to establish individual surveillance protocols.

In previous studies, we showed the convenience of adding EPCAM staining in the IHC algorithm approach for the screening of LS in CRC [12,13]. In a small series of 14 cases, we described that the complete loss of EPCAM expression in MSH2-negative CRC identified *EPCAM* 3′-end deletion carriers with 100% specificity. However, the limited sample size and the absence of additional results supporting these findings has prevented us from recommending the use of EPCAM IHC in daily clinical practice [18]. In this retrospective multicenter study, we increased the number of colonic cases. In addition, with the aim of knowing if the complete loss of EPCAM occurs in other tumors of the LS spectrum, we evaluated MSH2 and EPCAM expressions in a large series of malignant neoplasms, premalignant lesions of GI and extra-GI origin, and benign neoplasms located in different organs from patients with lack of MSH2 expression, in which we were able to perform a complete germ-line analysis. In addition, to extend the current knowledge of EPCAM expression, we evaluated the EPCAM expression by IHC in two tissue microarrays (TMAs), one constructed with normal tissues and the other with their malignant counterparts.

## 2. Results

### 2.1. Immunohistochemical Staining

One hundred and fifty-four (154/187; 82.3%) cases showed a loss of nuclear staining for MSH2, and the remaining 33 cases (33/187; 17.7%) were positive.

Only thirty-three (33/187; 17.7%) cases were EPCAM-negative, 17 (17/187; 9.1%) informative, and 16 (16/187; 8.5%) noninformative. The clinicopathological features of all series are detailed in Table S1.

#### 2.1.1. Malignant Neoplasms

**Figure 1:**
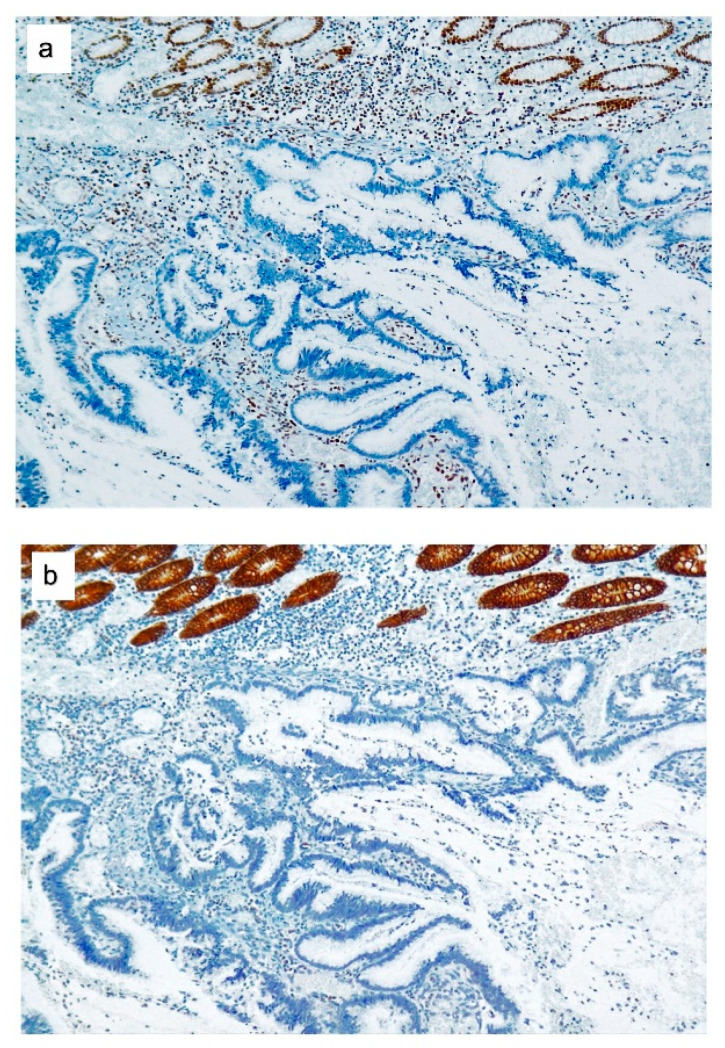
MSH2 and epithelial cell adhesion molecule (EPCAM) expressions in colorectal cancer (CRC) from *EPCAM* deletion carriers. (**a**) Loss of MSH2 expression in colorectal adenocarcinoma, mucinous type. (**b**) The same adenocarcinoma with a loss of EPCAM expression. Staining of normal colonic glands served as the internal positive control.

Ninety-four (94/99; 95%) malignant neoplasms were MSH2-negative (Figure 1), and only five (5/99; 5%) were MSH2-positive (Table 1).

Among the 99 malignant neoplasms, only 19 (19/99; 19.2%) showed a complete loss of EPCAM expression (Figure 1), and 10 (10/99; 10.1%) were informative and corresponded to CRC, all belonging to patients with *EPCAM* 3′-end deletions. The remaining nine (9/99; 9.1%) were considered noninformative. Of them, eight originated in tissues where EPCAM is not constitutively expressed, such as the squamous epithelia and the adrenal gland. The breast EPCAM-negative carcinoma was considered noninformative due to the inconsistent expression of EPCAM in this type of cancer (Table 1).

The distribution of both MSH2 and EPCAM expressions in malignant neoplasms showed statistical significance. However, only the expression of EPCAM had a relation of significance with the mutated genes (Table 1).

**Figure 2:**
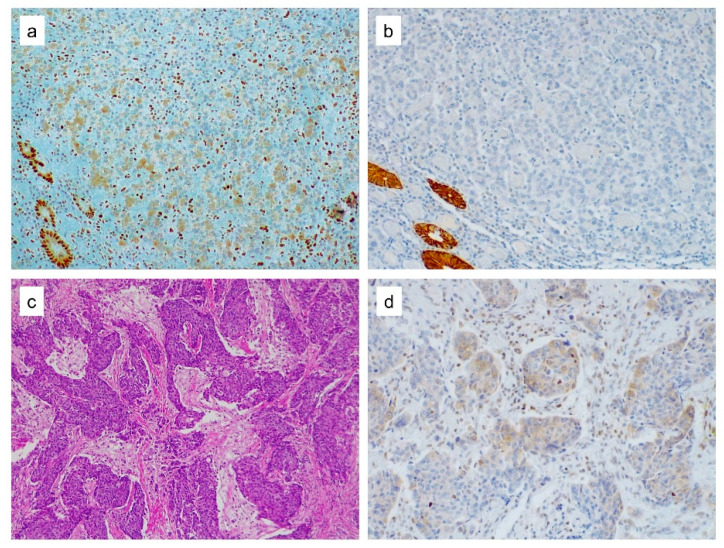
MSH2 cytoplasmic expression. (**a**) MSH2 cytoplasmic expression in both normal epithelial glands and in medullary colorectal cancer cells. Notice the lack of nuclear MSH2 expression in neoplastic cells. Lymphocytes in between the tumor cells showed nuclear staining and served as the internal positive control. (**b**) Loss of EPCAM expression in the tumor cells with normal colonic glands retaining the staining. (**c**) H&E staining of poorly differentiated gastric carcinoma displaying a cohesive solid pattern. (**d**) MSH2 staining yields a cytoplasmic expression in some of the tumor cells.

Two MSH2-negative malignant neoplasms showed also cytoplasmic staining, a mixed mucinous, and medullary carcinoma from the right colon and a poorly differentiated gastric carcinoma infiltrating the liver and the pancreas at diagnosis, both from patients of the same family with *EPCAM-MSH2* deletions (Figure 2).

#### 2.1.2. Precursor Lesions of GI Origin (Colorectal Polyps)

MSH2 expression was lost in 46 (46/71; 64.8%) colorectal polyps (Table 2). Distribution according to size, histology, and grade of dysplasia were significant. The majority of MSH2-negative colorectal polyps were tubulovillous adenoma (TVA) (100%) and traditional serrated adenoma (TSA) (100%), larger than 5 mm (33/46; 71.7%) and with high-grade dysplasia (HGD) (26/46; 56.5%).

**Figure 3:**
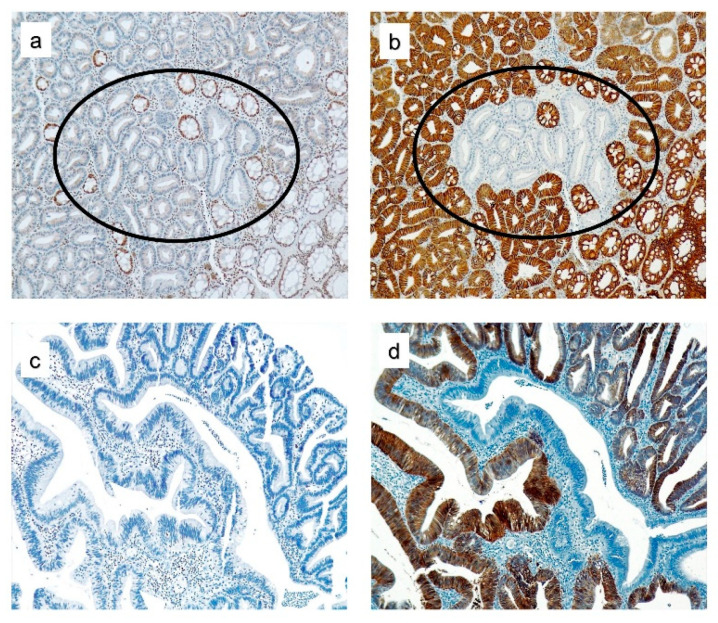
MSH2 and EPCAM expression in colorectal polyps from *EPCAM* deletion carriers. (**a**,**c**) Loss of MSH2 expression in colon adenomas. (**b**,**d**) Loss of EPCAM immunostaining only in isolated glands. Staining of normal colonic glands served as the internal positive control in (**a**,**b**), and (**d**). Lymphocytes cells were the internal positive control in (**c**).

A complete loss of EPCAM expression was observed in two colorectal polyps, while in the other five, the staining was lost only in isolated glands (Figure 3). All these lesions belonged to patients with *EPCAM* or *EPCAM-MSH2* deletions with statistical significance (Table 2). Clinicopathological features of the colorectal polyps are summarized in Table S2.

The relation between the mutated genes and the loss of expression of both MSH2 and EPCAM in colorectal polyps was significant.

#### 2.1.3. Precursor Lesions of Extra-GI Origin and Benign Neoplasms

Among nine precursor lesions, six (66.7%) were MSH2-negative endometrial hyperplasias. The three (33.3%) remaining were MSH2-positive, corresponding to two high squamous intraepithelial lesions (HSIL) and one ductal carcinoma in situ (DCIS) of the breast. All eight (100%) benign skin lesions were MSH2-negative (Table 3).

The 11 cases EPCAM-negative with MSH2 expression were considered noninformative: in the skin lesions and cervical HSIL, because there was no EPCAM staining in the normal squamous epithelia, and in the other of the breast because of the inconsistent expression of EPCAM in the breast tumor cells. The six endometrial hyperplasias maintained the expression of EPCAM (Table 3).

There was a significant association between the expression of MSH2 with the location and histology of the lesions. No statistical analysis was calculated for the EPCAM expression due to the negative EPCAM results being considered noninformative.

#### 2.1.4. Relation between MSH2 and EPCAM Immunostaining

The 154 MSH2-negative lesions corresponded to 121 EPCAM-positive (45 precursor lesions and 76 malignant neoplasms) and 33 EPCAM-negative; 17 from the colon were considered informative, and the other 16 cases (14 from the skin, 1 from the lip, and 1 adrenal carcinoma) were considered noninformative due to the lack of EPCAM staining in the normal tissue counterparts.

All 17 cases with a lack of staining of both MSH2 and EPCAM, which were informative, belonged to patients with *EPCAM* or *EPCAM-MSH2* deletions.

Among the 33 lesions with MSH2 expression, 29 were also EPCAM-positive (25 colorectal polyps and 4 carcinomas, 2 urothelial from the urinary bladder and 2 from the endometrium), and four were noninformative EPCAM-negative (two HSIL from the cervix and two lesions from the breast) (Table 4).

The relation between the MSH2 and EPCAM expressions was not statistically significant in all lesions (Table 4).

When we only analyzed the distribution of both the MSH2 and EPCAM expressions in the 43 lesions belonging to *EPCAM* 3′-end deletion carriers, only MSH2 was efficient at detecting these patients and showed a statistically significant relation (Table 5).

### 2.2. EPCAM Immunostaining in TMAs

#### 2.2.1. Normal Tissues

**Figure 4:**
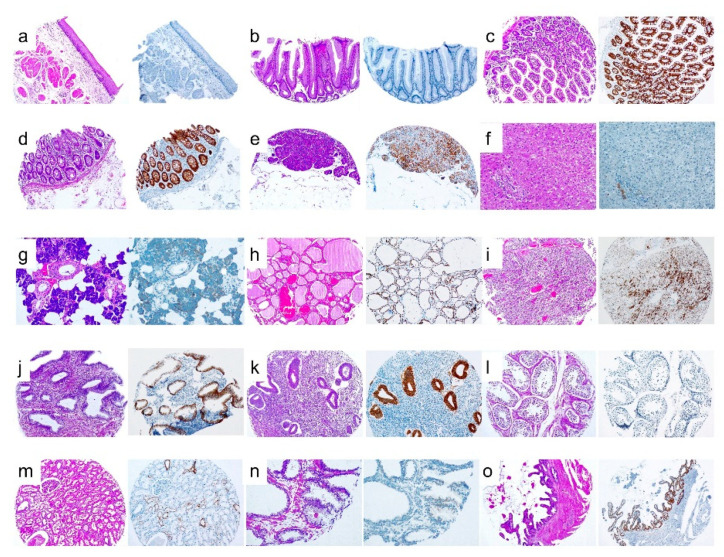
H&E staining and EPCAM expression in several normal tissues. (**a**) Esophagus, (**b**) stomach, (**c**) small bowel, (**d**) colon, (**e**) pancreas, (**f**) liver, (**g**) parotid, (**h**) thyroid, (**i**) parathyroid, (**j**) endocervix, (**k**) endometrium, (**l**) teste, (**m**) kidney, (**n**) prostate, and (**o**) seminal vesicle.

EPCAM was strongly expressed in the normal GI epithelia of the small bowel, colon, biliary tract, and in acinar pancreatic cells. EPCAM was also expressed in thyroid and parathyroid cells, endometrial and endocervical glands, the parotid, seminal vesicle epithelium, prostate and mammary epithelium, and in part of the epithelial cells of the kidney nephron (Table 6 and Figure 4). Lymphocytes, the central nervous system (CNS), and cells from soft tissues were EPCAM-negative.

#### 2.2.2. Tumor Tissues

The strongest EPCAM expression was seen in adenocarcinomas from the GI tract, i.e., small bowel, colon, pancreas, and cholangiocarcinomas but, also, in intestinal and diffuse gastric carcinomas, although gastric foveolar epithelium was EPCAM-negative. Endometrioid carcinoma from the endometrium and serous papillary and clear cell carcinomas from the ovary showed also strong EPCAM expressions. Neoplasms from the thyroid and parathyroid glands, squamous carcinoma, and adenocarcinomas from the lungs were EPCAM-positive. In the kidney, chromophobe carcinoma showed strong EPCAM expression, while clear cell renal cell carcinoma showed a weak staining. Prostate carcinoma, seminoma, yolk sack, and embryonic carcinomas from the testes showed partial positive staining. Low- and high-grade urothelial carcinomas displayed weak staining. Breast carcinomas showed partial staining that was less intense in the lobular type.

**Figure 5:**
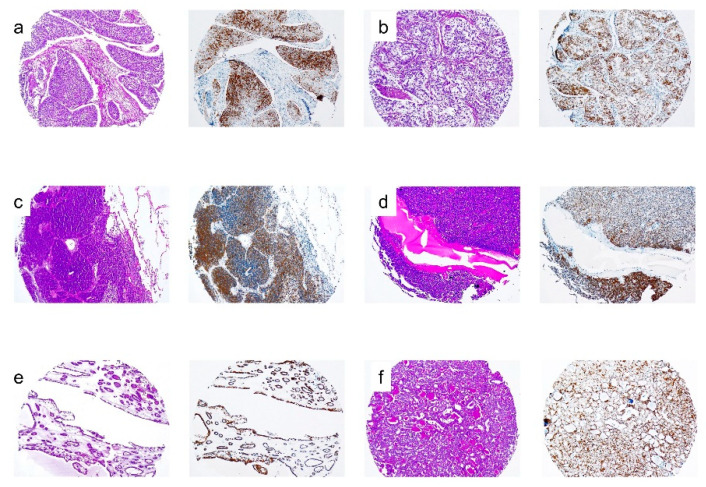
H&E staining and EPCAM expression in several tumor tissues. (**a**) Squamous lung carcinoma, (**b**) lung adenocarcinoma, (**c**) neuroendocrine lung carcinoma, (**d**) parathyroid adenoma, (**e**) thyroid adenoma, and (**f**) papillary thyroid carcinoma.

In addition to epithelial neoplasms, neuroendocrine carcinomas from the lungs and pancreas displayed EPCAM expression (Figure 5).

All tested lymphomas and tumors from the CNS and soft tissues were EPCAM-negative. All results are summarized in Table 6.

### 2.3. Germ-Line Analysis

LS was confirmed in 68 patients, 53 with pathogenic variants in *MSH2* gene, 13 with deletion of exons 8 and 9 of *EPCAM* from five families, and 2 with *EPCAM-MSH2* deletions from one family. One patient (#69) was a carrier of a variant of unknown significance in the *MSH2* gene and in another two (#70 and #71), no mutations were found (Table S1).

### 2.4. Distribution of MSH2 and EPCAM Expression According to Germ-Line-Mutated Genes

#### 2.4.1. MSH2 and EPCAM Expression in Lesions from All Patients

MSH2 was positive in 29 (29/142; 20.4%) of 142 lesions from patients with *MSH2* pathogenic variants. The relation between the expression of MSH2 and the mutated genes was not significant (Table 7).

The 17 (17/43; 39.5%) informative EPCAM-negative lesions belonged to patients with *EPCAM*-3′-end deletions. None of the 124 cases with *MSH2* pathogenic variants showed a loss of EPCAM expression. The association between the expression of EPCAM and the mutated genes was statistically significant (Table 7).

The sensitivity of the loss of MSH2 expression to identify *EPCAM*-3′ deletion carriers was 90.6% (34 + 5/[34 + 5) + 4] ×100), while the specificity was 20.4% (29/(29 + 113) ×100). On the contrary, the sensitivity of the loss of EPCAM expression to identify *EPCAM*-3′ deletion carriers was 41.4% (15 + 2/[(15 + 2) + (21 + 3)] ×100), while the specificity reached 100% (124/(124 + 0) ×100).

#### 2.4.2. MSH2 and EPCAM Expression in Lesions from EPCAM-3′-End Deletion Carriers

Thirty-nine (39/43; 90.6%) of the 43 lesions from patients with *EPCAM*-3′-end deletions were MSH2-negative. The expression of MSH2 was efficient in detecting lesions from patients with both *EPCAM* and *EPCAM*-*MSH2* pathogenic variants with statistical significance (Table 8).

Of the 43 lesions from patients with *EPCAM* deletions, 17 (17/43; 39.5%) cases were informatively EPCAM-negative: 7 colorectal polyps and 10 CRC. In all cases, EPCAM staining was present in adjacent normal tissues. All lesions with an informative loss of EPCAM expression belonged to patients with *EPCAM* (15/17) or *EPCAM-MSH2* (2/17) deletions. In 20 cases (20/187; 10.7%), the lack of EPCAM staining was considered noninformative. The expression of EPCAM was not efficient at detecting lesions from patients with both *EPCAM* and *EPCAM-MSH2* pathogenic variants. No statistical association was found between the expression of EPCAM and the mutated genes (Table 8).

## 3. Discussion

EPCAM immunostaining is a very useful tool to guide the germ-line analysis of LS in MSH2-negative colorectal malignant neoplasms and MSH2-negative colorectal polyps. Considering that the LS tumor spectrum is different according to the involved gene, it is crucial to know the specific germ-line alteration to establish individual surveillance protocols [19].

### 3.1. MSH2 Expression

A total of 68 out of 71 patients were carriers of pathogenic variants in *EPCAM* and/or *MSH2* genes. Thus, it was expected that all malignant neoplasms were MSH2-negative. However, six malignant neoplasms displayed MSH2 expression, probably as a result of the activation of other carcinogenetic pathways. MSH2 expression in one cervical precursor lesion and in one urothelial carcinoma of the urinary bladder could be explained by the oncogenic role of human papillomavirus and tobacco as potent carcinogens in the cervix and urothelial epithelium [20,21]. In the two endometrial MSH2-positive cancers, the remaining MMR proteins were also expressed, although the PCR microsatellite instability analysis results were unstable (data not shown), consistent with results previously reported [22,23]. In the two breast lesions, other carcinogenetic pathways different from the MMR system may be activated [24].

#### 3.1.1. MSH2 in Precursor Lesions of GI Origin (Colorectal Polyps)

We showed that 86% of colorectal polyps were MSH2-negative, adequate for the identification of LS mutation carriers. Those deficient colorectal polyps presented more frequently in older patients (≥60 years) were larger than 5 mm, with a histology of TSA and TVA, and were located in the left colon and rectum and displayed HGD. Only size, histology, and dysplasia were statistically significant. Our results support previous studies [25,26,27,28,29,30,31,32,33,34,35,36] and demonstrate that colorectal polyps are a valuable sample that allows identifying LS mutation carriers when no carcinoma tumor sample is available, especially if larger than 5 mm, in TVA or TSA with HGD.

#### 3.1.2. Aberrant Cytoplasmic MSH2 Expression

In addition to the loss of nuclear MSH2 expression in malignant neoplasms, aberrant cytoplasmic immunoreactivity was observed in a mixed mucinous and medullary CRC, the normal colonic mucosa counterpart, and in a poorly differentiated gastric carcinoma from patients of family 5 (#12 and #13). The study of large germ-line rearrangements in *EPCAM* of patient #12 showed a heterozygous deletion of a region of 15 Kb, which included exons 8 and 9 of *EPCAM*, located 2.5-Kb upstream from the start codon of *MSH2*. The analyzed CRC showed methylation of the promoter region of *MSH2*, which would be induced by the large deletion of the *EPCAM*-intergenic region *EPCAM-MSH2* and would explain the absence of MSH2 protein expression. The same patient had uterine cancer, but a tumor sample was not available. To our best knowledge, there were only two previous cases reported in the literature with cytoplasmic MSH2 IHC patterns, both belonging to LS patients: a colon adenoma [37] and a medullary CRC [38]. In the latter, the aberrant immunoreactivity in both colon cancer and normal mucosa was explained by the existence of an *EPCAM-MSH2* fusion transcript. In addition, the fusion transcript should contain premature stop codons within *MSH2*, resulting in the lack of nuclear staining in tumor cells [38].

Although neoplasms from patients #12 and #13 shared the same pathogenic variant, they showed different EPCAM expressions. Only the CRC from patient #12 had a biallelic *EPCAM* deletion that correlated with the lack of expression (see below). Of interest, all cases described with MSH2 cytoplasmic staining and loss of nuclear staining were from the GI epithelia. Therefore, this IHC pattern in an adenoma or CRC could be highly suggestive of a concomitant *EPCAM* deletion and a useful feature to remember.

### 3.2. EPCAM Expression

#### 3.2.1. EPCAM Tumor Spectrum

Homozygote mutation of *EPCAM* causes congenital tufting enteropathy, affecting the intestinal epithelium with severe diarrhea in newborns [39,40]. Heterozygote deletions of the *EPCAM* 3′ end in germ cells account for up to 2% to 3% LS cases [7,16] due to different mechanisms. One is the exclusive deletion of the 3′ extreme of *EPCAM*. In the other, the *EPCAM* 3′-end deletion extends to the first exons of the 5′ end of MSH2, including the promoter region. In each case, the tumor spectrum is different and varies in relation to the size and location of the *EPCAM* 3′-end deleted fragment [16].

Lynch et al. and Grandval et al., using large families with *EPCAM* 3′-end deletions carriers, described malignant neoplasms only from the GI tract, being CRC the most frequent [41,42]. Among our eleven patients with *EPCAM* deletions in exon 9, only one presented a duodenal carcinoma, while the remainder corresponded to CRC, corroborating these findings.

Kempers et al., analyzing 194 carriers with *EPCAM* 3′-end deletions, reported endometrial carcinomas with a life risk of 12% (0–27) lower than obtained in *MSH2* and *MSH6* mutation carries (51% (33–69) and 34% (20–48), respectively), although ascertainment bias led to an overestimation of cancer risk in all the groups [16]. The authors correlated the type of neoplasm with the size and location of the deleted region in the *EPCAM* gene. A higher risk of extra-GI tumors was observed in deletions that extended close to the *MSH2* promoter region [16]. Our study corroborates Kempers’ observations, specifically patient #12 from family 5, the carrier of an *EPCAM* deletion of exons 8 and 9 extending very close to the promotor region of *MSH2*, who presented both colorectal and endometrial carcinomas. In our series, other gynecological neoplasms appeared in *EPCAM-MSH2* deletions carries—mostly, endometrial carcinoma—confirming the observations of previous studies [16,17]. However, as far as we know, it is the first time that a clear cell ovarian carcinoma is reported in this subset of *EPCAM-MSH2* LS patients.

Although it needs to be confirmed by larger collaboration studies, the low incidence of extra-GI neoplasms in the spectrum of *EPCAM* LS tumors could be, in part, explained by the mechanism of epigenetic *MSH2* silencing. *MSH2* inactivation is allele-specific and involves the allele segregating with the *EPCAM* deletion. This explains why *MSH2* methylation is restricted to EPCAM-positive normal cells [8]. Therefore, the high expression of EPCAM in colonic stem cells justifies the high incidence of CRC in patients with *EPCAM* 3′-end deletions. Surprisingly, despite the high EPCAM expression in thyroid and neuroendocrine tissues, tumors from these origins are not present in *EPCAM* 3′-end deletion carriers. These neoplasms are neither part of the LS tumor spectrum due to *MSH2* alterations, confirming the important role that *MSH2* plays in tumor phenotypes of patients with *EPCAM* deletions.

#### 3.2.2. EPCAM Expression in TMAs

The EPCAM protein is a transmembrane type I glycoprotein located in the basolateral membrane of normal epithelial cells, except in hepatocytes, thymic cortical epithelial cells, squamous epithelia, epidermal keratinocytes, gastric parietal cells, and myoepithelial cells [43]. Using TMAs constructed with different normal and neoplastic tissues, we observed that EPCAM expression in neoplasms reflected the expression of its normal counterpart. Thus, adenocarcinomas from the GI tract and endometrium were strongly positive. Thyroid and neuroendocrine neoplasms also displayed EPCAM expression, but squamous carcinoma, hepatocellular tumors, lymphomas, the CNS, and soft tissue tumors were EPCAM-negative. However, gastric and lung carcinomas showed EPCAM expression, despite the lack of staining in normal gastric foveolar epithelium and lung parenchyma. Contrarily, normal breast epithelium was EPCAM-positive, but the carcinomas displayed a focal and weak staining, especially the lobular type, as reported by Spizzo et al. [44].

In view of these results, the loss of EPCAM expression in those neoplasms where EPCAM was not expressed in their normal counterpart was considered noninformative. The lack of expression of EPCAM in neoplasms from *EPCAM* 3′-end carriers will be more significant as the more intense and robust the expression is in its normal tissue. Therefore, a negative EPCAM staining in renal, urothelial, and breast cancers should be evaluated with caution, mostly in the presence of a concomitant negative MSH2 staining.

#### 3.2.3. EPCAM Expression in Malignant Neoplasms

EPCAM protein was expressed in adenocarcinomas of the GI tract, especially in CRC, where it showed a diffuse and strong positivity. The fact that the absence of EPCAM expression identifies patients with *EPCAM* 3′-end deletions has already been demonstrated [10,11]. In our series, CRC was the most informative tumor, being the unique neoplasm that showed a complete loss of EPCAM expression, identifying patients with *EPCAM* 3′-end deletions with 100% specificity and confirming our previous results [12,13]. A partial loss of EPCAM staining in CRC was described in poorly differentiated carcinomas and/or at the invasive tumor front as an independent poor prognostic factor in nondeficient MMR CRC [15]. However, not all CRC from patients with *EPCAM* 3′-end deletions showed a lack of EPCAM expression. Only when the second somatic hit affects the *EPCAM* gene, resulting in a biallelic *EPCAM* 3′-end deletion, a lack of EPCAM expression will be observed [11]. This explains why our patients #5 and #6 with synchronic CRCs displayed different EPCAM expression in their carcinomas. The two gynecological MSH2-negative neoplasms in carriers with double-*EPCAM-MSH2* deletions retained EPCAM staining, despite the intense EPCAM expression observed in the normal endometrial mucosa. Further gynecological tumors from *EPCAM* 3′-end deletion carriers should be tested to evaluate the usefulness of EPCAM IHC in endometrial carcinomas.

A complete loss of EPCAM expression strongly correlates with *EPCAM* biallelic 3′-end deletion and *MSH2* silencing only in those tissues where EPCAM is expressed in their normal counterparts.

#### 3.2.4. EPCAM Expression in Precursor Lesions of GI Origin (Colorectal Polyps)

The use of colorectal polyps in LS screening is justified when no tumor tissue is available. In this scenario, adding EPCAM IHC to MSH2-negative colorectal polyps provides useful information. As Huth et al. described previously, a loss of EPCAM staining was observed in colorectal polyps from *EPCAM* 3′-end deletion carriers [11]. In our series, five of seven colorectal polyps showed a lack of EPCAM expression in isolated glands belonging to patients with both *EPCAM* 3′-end deletions and combined *EPCAM-MSH2* deletions. This feature was not observed in MSH2-negative colorectal polyps from patients with other pathogenic variants. A focal loss of MMR protein in colonic adenomas of LS patients is rare [37,45], and even more exceptional is to find deficient isolated colonic crypts [46,47]. However, a partial loss of EPCAM staining is frequent in *EPCAM* 3′-end deletion carriers and helps to recognize this pathogenic variant.

Our study had some limitations, such as the low number of extra-GI primary neoplasms in *EPCAM* deletion carriers, especially endometrial carcinoma, not allowing to know the usefulness of EPCAM IHC in this type of carcinomas. We want to mention that, currently, new technologies such as next-generation sequencing (NGS) make more accessible the diagnosis of LS. The germ-line analysis of patients with MSH2-negative malignant neoplasms uses NGS and an exon-level array comparative genomic hybridization–based or multiplex ligation-dependent probe amplification (MLPA)-based deletion/duplication analysis of all exons and adjacent noncoding regions [48]. With this entire arsenal, an *EPCAM* analysis is included guaranteeing the identification of alterations affecting this gene, which could make less relevant the inclusion of EPCAM IHC in the screening algorithm. Nevertheless, an IHC analysis is cheap, fast, and well-incorporated in the daily practice routine of all pathology departments. Adding EPCAM immunostaining to the IHC screening algorithm for LS patient detection when MSH2 is negative improves the results.

## 4. Materials and Methods

The present study aims to analyze the role of IHC staining of EPCAM in the screening algorithm of LS.

For this purpose, we designed a retrospective and multicenter study with the participation of several hospitals where IHC screening of LS in colorectal and endometrial carcinoma is routinely performed in pathology departments. The participation of the Genetic Counseling Units helped to identify lesions belonging to patients with LS due to pathogenic variants of *EPCAM*.

The study series consisted of malignant neoplasms, precursor lesions of gastrointestinal and extra gastrointestinal origin, and benign neoplasms of various organs. The expression of MSH2 and EPCAM was analyzed by IHC staining, and germ-line analysis was performed.

The results obtained were analyzed with statistical methods. We elaborated the conclusions with the data obtained with statistical significance (*p* < 0.05).

### 4.1. Cases

In this multicenter and retrospective study, we collected a total of 190 lesions according to the following inclusion criteria: (a) those with a lack of MSH2 expression belonging to patients in whom we were able to perform a complete germ-line analysis for *MSH2* gene alterations. In this way, we collected the most of the malignant neoplasms from the files of the hospitals participating in the study. (b) Lesions belonging to patients with LS due to *EPCAM* or *MSH2* pathogenic variants from the files of the Genetic Counseling Units. This way, it was possible to increase the number of lesions belonging to carriers with pathogenic variants of *EPCAM*. The series was recruited from April 2018 to February 2019.

Patients were 43 (61%) females and 28 (39%) males, and ages ranged between 24 and 82 years (median age 50.8 years). LS was confirmed in 68 patients: 15 had deletions affecting the 3′ end of *EPCAM*; in 2 of them, the deletion also involved the 5′ end of *MSH2* (*EPCAM-MSH2* deletion); 53 patients had pathogenic mutations in the *MSH2* gene, and 3 were LS-like patients: 1 with a variant of unknown significance and 2 with no mutation found. CRC from family 1 were included in previous reports [12,13].

Regarding location, 134 (70.6%) lesions developed in the GI tract (2 in the duodenum, 1 in the stomach, 1 in the pancreas, 1 in the appendix, 60 in the right colon, 20 in the left colon, 19 in the rectum, and 30 without specific location); 28 (14.8%) in the female reproductive system (2 in the cervix, 2 in the ovary, and 24 in the endometrium); 14 (7.3%) in the skin and 1 (0.5%) in the lip; 7 (3.7%) in the urinary tract (1 in the ureter, 1 in the renal pelvis, and 5 in the urinary bladder); 2 (1%) in the breast; 1 (0.5%) in the prostate; and 1 (0.5%) in the adrenal gland. Only in 2 (1%) cases, the origin of the neoplasm was unknown. In 3 cases, the tumor samples were not available.

Histologically, 8 (4.2%) lesions were benign, 9 (4.7%) were precursor lesions of extra-GI origin, 71 (37.3%) were precursor lesions of GI origin (colorectal polyps), and 102 (53.8%) malignant neoplasms. Malignant neoplasms belonged to 64 patients: 37 (58%) females and 27 (42%) males, with a median age at diagnosis of 48 years.

A total of 46 lesions belonging to 15 patients from 6 families with germ-line *EPCAM* 3′-end deletions were described. Among malignant neoplasms, the majority were from the GI tract, especially CRC, but also from the stomach, duodenum, and pancreas. There were 4 extra-GI neoplasms: 2 endometrial carcinomas, 1 clear cell carcinoma from the ovary, and 1 Hodgkin’s lymphoma.

There were 71 colorectal polyps from 27 patients: 15 females and 12 males from ages ranging 24 to 81 years (median age 53.1 years). Fifty-three colorectal polyps belonged to 20 patients with *MSH2* pathogenic variants: 15 with *EPCAM* 3′-end deletions and 3 with *EPCAM-MSH2* deletions. According to location, 1 (1.4%) was in the appendix, 36 (50.7) in the right colon, 12 (16.9%) in the left colon, 13 (18.3%) in the rectum, and in 9 (12.7%) cases, the exact location was unknown. Thirty (42.3%) polyps were equal or smaller than 5 mm, 28 (39.4%) between 6 and 10 mm, and 13 (18.3%) equal or larger than 11 mm. Histologically, 4 (5.6%) were hyperplastic polyps (HP), 35 (49.3%) tubular adenomas (TA), 26 (36.6%) sessile serrated lesions (SSL), 4 (5.6%) TSA, and 2 (2.8%) TVA. Thirty-three (46.5%) polyps showed HGD (14 TA, 13 SSL, 4 TSA, and 2 TVA) and 28 (39.4%) LGD. In the remaining 10 (14.1%), no dysplasia was found (Table 2).

Informed consent was obtained from all patients included in the study. As a retrospective study, if patients had died, consent was by death. The rest of the patients were contacted to obtain an informed consent in accordance with the Ley 14/2007 de Investigación Biomédica (B.O.E. 159 4 July 2007). Some patients signed an informed consent form that diagnostic surplus material could be used for research. All patients signed an informed consent form for germ-line analysis. The study was approved by the Institutional Ethics Committee for Clinical Research (CEIC) under code 2017/57-APA-HUGC.

### 4.2. Tissue Microarrays

We constructed 2 TMAs, one with a collection of 34 different normal tissues and the other with 57 distinct neoplastic tissues. Three cylindrical cores, each measuring 0.6 mm in diameter, were obtained from every donor paraffin block using a tissue microarray workstation MTA-1 (Beecher Instruments, Silver Spring, MD, USA). EPCAM expression was first independently evaluated by each of the two authors (M.C. and E.M.) and then jointly reevaluated under a double-headed microscope for final score agreement.

### 4.3. Immunohistochemistry

Formalin-fixed, paraffin-embedded tissue sections were analyzed using standard IHC techniques. Immunostaining was performed automatically using a Ventana BenchMark ULTRA machine (Roche, Basel, Switzerland). The mouse primary antibodies used were anti-hMSH2 (clone G219-1129, Ventana Medical Systems, Inc., 1910 E. Innovation Park Drive, Tucson, AZ 85755, USA) and anti-EPCAM antibody (clone Ber-EP4, Cell Marque Corporation, 6600 Sierra College Blvd., Rocklin, CA 95677, USA). Positive staining for the anti-hMSH2 antibody was located in the nucleus of the neoplastic cells. Nuclear immunoreaction in lymphocytes, normal colonic mucosa, or stromal cells served as the internal anti-hMSH2 positive control. Loss of MSH2 staining was considered when the nuclei of all neoplastic cells were negative. Positive staining for the anti-EPCAM antibody was located in the membrane of the neoplastic epithelial cells. Membrane immunostaining in normal colonic mucosa was used as the internal anti-EPCAM positive control. Complete loss of EPCAM staining was only considered when the total of the neoplastic cells was completely negative. We considered it as informative staining when the normal tissue was positive and as noninformative when the normal counterpart was negative or when the staining in the tumor was not strong and homogeneous. MSH2 and EPCAM immunostaining were independently evaluated by two expert pathologists (M.C. and E.M.). The slides were anonymized and were interpreted without knowing the germ-line results.

### 4.4. Germ-Line Mutations

Germ-line mutation studies were performed on genomic DNA isolated from peripheral blood leucocytes in different laboratories using sequencing and MLPA techniques. MMR variant classification was determined according to InSIGHT classification guidelines [49].

### 4.5. Statistical Analysis

Analysis was carried out using SSPS software version 15.0 (SPSS, Chicago, IL, USA). The χ^2^ test was used to analyze the association between qualitative variables, followed by the Fisher’s exact test and the Student’s *t*-test or the Mann-Whitney test for quantitative variables. A *p* < 0.05 was considered significant.

## 5. Conclusions

In summary, we demonstrate that EPCAM IHC is a useful tool that contributes to identify LS patients with *EPCAM* 3′-end deletions in the screening of MSH2-negative CRC. Thus, we recommend adding EPCAM IHC to the algorithm approach for LS identification in MSH2-negative CRC, where the absence of EPCAM expression reaches 100% specificity.

In addition, the presence of isolated EPCAM-negative glands in MSH2-negative colorectal polyps is a hallmark pattern that allows the identification of *EPCAM* 3′-end deletion carriers, demonstrating its effectiveness when no tumor tissue is available.

## Figures and Tables

**Table 1:** MSH2 and epithelial cell adhesion molecule (EPCAM) expression in malignant neoplasms.

Malignant Neoplasms	MSH2 Expression			*p*-Value	EPCAM Expression				*p*-Value
	−	+	Total		−		+	Total	
					I	NI			
Location				0.000					0.000
GI									
Stomach	1	0			0	0	1		
Duodenum	2	0			0	0	2		
Colon, unknown	20	0			4	0	16		
Right colon	24	0			5	0	19		
Left colon	8	0			1	0	7		
Rectum	6	0			0	0	6		
Pancreas	1	0			0	0	1		
Endometrium	15	2			0	0	17		
Skin	6	0			0	6	0		
Urinary bladder	3	2			0	0	5		
Ovary	2	0			0	0	2		
Lip	1	0			0	1	0		
Breast	0	1			0	1	0		
Adrenal gland	1	0			0	1	0		
Renal Pelvis	1	0			0	0	1		
Ureter	1	0			0	0	1		
Prostate	1	0			0	0	1		
Unknown	1	0			0	0	1		
	94	5	99		10	9	80	99	
Mutated gene				0.62					0.003
*EPCAM*	21	0			10		11		
*EPCAM-MSH2*	2	0			0		2		
*MSH2*	69	5			9		65		
Unknown	2	0			0		2		
	94	5	99		19		80	99	

Abbreviations: −, negative; +, positive; I, informative; NI, noninformative; and GI, gastrointestinal.

**Table 2:** Clinicopathological features of colorectal polyps according to expression of MSH2 and EPCAM.

Colorectal Polyps	MSH2 Expression			*p*-Value	EPCAM Expression					*p*-Value
	−	+	Total		−			+	Total	
					I		NI			
					CL	FL				
Location				0.178						0.08
Right colon	21	16			1	0	0	36		
Left colon and rectum	17	8			1	4	0	20		
Unknown	8	1			0	1	0	8		
	46	25	71		2	5	0	64	71	
Size (mm)				0.020						0.597
≤5	14	16			0	2	0	28		
6-10	21	7			1	3	0	24		
≥11	11	2			1	0	0	12		
	46	25	71		2	5	0	64	71	
Histology				0.018						0.766
TA	25	10			1	2	0	32		
TVA	2	0			0	0	0	2		
HP	0	4			0	0	0	4		
SSL	15	11			1	2	0	23		
TSA	4	0			0	1	0	3		
	46	25	71		2	5	0	64	71	
Dysplasia				0.000						0.086
WD	1	9			0	0	0	10		
LGD	19	9			0	1	0	27		
HGD	26	7			2	4	0	27		
	46	25	71		2	5	0	64	71	
Mutated gene				0.042						0.000
*EPCAM*	13	2			1	4	0	10		
*EPCAM-MSH2*	3	0			1	1	0	1		
*MSH2*	30	23			0	0	0	53		
	46	25	71		2	5	0	64	71	

Abbreviations: −, negative; +, positive; I, informative; NI, noninformative; CL, complete loss; FL, focal loss; TA, tubular adenoma; TVA, tubulovillous adenoma; HP, hyperplastic polyp; SSL, sessile serrated lesion; TSA, traditional serrated adenoma; WD, without dysplasia; LGD, low-grade dysplasia; and HGD, high-grade dysplasia.

**Table 3:** Clinicopathological features of precursor lesions of extra-GI origin and benign neoplasms according to the expressions of MSH2 and EPCAM.

	MSH2 Expression			*p*-Value	EPCAM Expression				*p*-Value
	−	+	Total		−		+	Total	
					I	NI			
Lesion									
Benign	8	0			0	8	0		
Precursor extra GI	6	3			0	3	6		
	14	3	17		0	11	6	17	
Location				0.000					
Skin	8	0			0	8	0		
Breast	0	1			0	1	0		
Cervix	0	2			0	2	0		
Endometrium	6	0			0	0	6		
	14	3	17		0	11	6	17	
Histology				0.004					
Sebaceoma	3	0			0	3	0		
Sebaceous adenoma	5	0			0	5	0		
DCIS	0	1			0	1	0		
HSIL	0	2			0	2	0		
SH	2	0			0	0	2		
CH	4	0			0	0	4		
	14	3	17		0	11	6	17	
Mutated gene				0.22					
*MSH2*	14	1			0	9	6		
*EPCAM*	0	2			0	2	0		
	14	3	17		0	11	6	17	

Abbreviations: −, negative; +, positive; I, informative; NI, noninformative; DCIS, ductal carcinoma in situ; HSIL, high squamous intraepithelial lesion; SH, simplex hyperplasia; and CH, complex hyperplasia.

**Table 4:** Relation between MSH2 and EPCAM expressions.

		EPCAM Expression				*p*-Value
		−		+	Total	
		I	NI			
		17	16			0.165
MSH2 expression	-	33		121	154	
		0	4			
	+	4		29	33	
	Total	37		150	187	

Abbreviations: −, negative; +, positive; I, informative; and NI, noninformative.

**Table 5:** Distribution of MSH2 and EPCAM expressions in lesions from *EPCAM* 3′-end deletion carriers.

Lesions From *EPCAM* 3′-End Deletion Carriers	MSH2 Expression			*p*-Value	EPCAM Expression					*p*-Value
	(−)	(+)	Total				(−)	(+)	Total	
					I		NI			
					CL	FL				
				0.000						0.510 *
Malignant neoplasms	23	0			10	0	0	13		
Colorectal polyps	16	2			2	5	0	11		
Precursor lesions extra-GI origin	0	2			0	0	2	0		
	39	4	43		12	5	2	24	43	

Abbreviations: −, negative; +, positive; I, informative; NI, noninformative; CL, complete loss; and FL, focal loss. * EPCAM *p*-value was calculated on 41 cases, because noninformative cervical lesions were eliminated.

**Table 6:** EPCAM expression in tissue microarray samples.

Normal Tissues		EPCAM Expression	Tumor Tissues		EPCAM Expression
1	Parotid	+ weak	1	Pleomorphic adenoma	−
			2	Myoepithelioma	−
			3	Mucoepidermoid carcinoma	+
2	Thyroid	+	4	Papillary carcinoma	+
			5	Follicular adenoma	+
			6	Medullary carcinoma	−
3	Parathyroid	+	7	Parathyroid adenoma	+
4	Lung #	−	8	Squamous cell lung carcinoma	+
			9	Lung adenocarcinoma	+
			10	Bronchial-alveolar carcinoma	+
			11	Lung neuroendocrine carcinoma	+
5	Lymph node	−	12	Large cell diffuse B-cell lymphoma	−
			13	Chronic lymphocytic leukemia	−
			14	Follicular lymphoma	−
			15	Mantle lymphoma	−
			16	Peripheral T lymphoma	−
			17	T angioimmunoblastic lymphoma	−
6	Thymus	−	18	Thymoma	−
7	Esophagus	−	19	Squamous cell esophageal carcinoma	−
8	Stomach α	−	20	Intestinal gastric adenocarcinoma	+
			21	Diffuse gastric adenocarcinoma	+
9	Small bowel	+	22	Carcinoma	+
10	Colon	+	23	Colon adenocarcinoma	+
11	Liver β	−	24	Hepatic adenoma	−
			25	Hepatocellular carcinoma	−
			26	Cholangiocarcinoma	+
12	Pancreas δ	+	27	Pancreatic ductal adenocarcinoma	+
			28	Neuroendocrine carcinoma	+
13	Kidney ε	+	29	Renal clear cell carcinoma	+ weak
			30	Chromophobe renal cell carcinoma	+
			31	Kidney oncocytoma	−
14	Adrenal gland	−	32	Adrenal adenoma	−
			33	Adrenal carcinoma	−
			34	Pheochromocytoma	−
15	Prostate	+	35	Prostate adenocarcinoma	+
16	Testis	−	36	Testicular seminoma	+
			37	Testicular yolk sac carcinoma	+
			38	Testicular embryonic carcinoma	+
17	Endometrium	+	39	Endometrioid carcinoma	+
18	Endocervix	+	40	Squamous cell carcinoma of cervix	−
19	Ovary	-	41	Serous papillary carcinoma	+
			42	Clear cell carcinoma	+
20	Urinary bladder	−	43	Low-grade urothelial carcinoma	+ weak
			44	High-grade urothelial carcinoma	+ weak
21	Breast	+	45	Fibroadenoma	+
			46	Ductal carcinoma	+ weak
			47	Lobular carcinoma	+ weak
22	Skin	−	48	Skin melanoma	−
			49	Merkel cell carcinoma	−
			50	Kaposi’s sarcoma	−
23	Skeletal muscle		51	Rhabdomyosarcoma	−
24	Smooth muscle		52	Leiomyoma	−
			53	Leiomyosarcoma	−
	Others			Others	
25	White matter	−	54	Malignant mesothelioma	−
26	Gray matter	−	55	Pituitary adenoma	−
27	Cerebellum	−	56	Synovial sarcoma	−
28	Palatine tonsil	−	57	Schwannoma peripheral nerve	−
29	Spleen	−			
30	Myocardium	−			
31	Placenta	−			
32	Adipose tissue	−			
33	Seminal vesicle	+			
34	Aorta	−			

Abbreviations: #, alveoli and bronchial epithelium; α, foveolar epithelium; β, negative hepatocytes and positive biliary epithelium; δ, acinar cells; and ε, focal positivity in part of the nephron.

**Table 7:** Distribution of MSH2 and EPCAM expressions according to the germ-line mutated genes in lesions from all patients.

Mutated Genes	MSH2 Expression			*p*-Value	EPCAM Expression				*p*-Value
	-	+	Total		−		+	Total	
					I	NI			
				0.311					0.000 *
*EPCAM*	34	4	38		15	2	21	38	
*EPCAM-MSH2*	5	0	5		2	0	3	5	
*MSH2*	113	29	142		0	18	124	142	
Unknown	2	0	2		0	0	2	2	
	154	33	187		17	20	150	187	

Abbreviations: −, negative; +, positive; I, informative; and NI, noninformative. * EPCAM *p*-value was calculated on 167 cases, because noninformative lesions were eliminated.

**Table 8:** Distribution of MSH2 and EPCAM expressions in lesions from *EPCAM*3′-end deletion carriers.

Lesion From *EPCAM* 3′-End Deletion Carriers	MSH2 Expression			*p*-Value	EPCAM Expression					*p*-Value *
	(−)	(+)	Total				(−)	(+)	Total	
					I		NI			
					CL	FL				
				0.000						0.510 *
Malignant neoplasms										
Location										
Stomach	1	0			0	0	0	1		
Duodenum	1	0			0	0	0	1		
Colon and rectum	19	0			10	0	0	9		
Endometrium	1	0			0	0	0	1		
Ovary	1	0			0	0	0	1		
	23	0	23		10	0	0	13	23	
Colorectal polyps										
Histology										
TA	6	0			1	2	0	3		
TVA	1	0			0	0	0	1		
HP	0	2			0	0	0	2		
SSL	7	0			1	2	0	4		
TSA	2	0			0	1	0	1		
	16	2	18		2	5	0	11	18	
Precursor lesions extra-GI origin										
Location										
Cervix	0	2	2		0	0	2	0	2	
			43						43	
Mutated gene				0.598						0.665 *
*EPCAM*	34	4			15	0	0	21		
*EPCAM-MSH2*	5	0			2	0	0	3		
	39	4	43		17	0	0	24	43	

Abbreviations: −, negative; +, positive; I, informative; and NI, noninformative. * EPCAM *p*-value was calculated on 41 cases, because noninformative cervical lesions were eliminated.

## Supplementary Materials

The following are available online at https://www.mdpi.com/2072-6694/12/10/2803/s1, Table S1: Clinicopathological features and molecular alterations of all cases, Table S2: Clinicopathological features and molecular alterations of colorectal polyps.

## References

[1] Lynch H.T., Snyder C.L., Shaw T.G., Heinen C.D., Hitchins M.P. (2015). Milestones of Lynch syndrome: 1895–2015. Nat. Rev. Cancer.

[2] Tiwari A.K., Roy H.K., Lynch H.T. (2016). Lynch syndrome in the 21st century: Clinical perspectives. QJM.

[3] Giardiello F.M., Allen J.I., Axilbund J.E., Boland C.R., Burke C.A., Burt R.W., Church J.M., Dominitz J.A., Johnson D.A., Kaltenbach T. (2014). Guidelines on genetic evaluation and management of Lynch syndrome: A consensus statement by the US Multi-Society Task Force on colorectal cancer. Gastroenterology.

[4] Kovacs M.E., Papp J., Szentirmay Z., Otto S., Olah E. (2009). Deletions removing the last exon of *TACSTD1* constitute a distinct class of mutations predisposing to Lynch syndrome. Hum. Mutat..

[5] Jasperson K.W., Tuohy T.M., Neklason D.W., Burt R.W. (2010). Hereditary and familial colon cancer. Gastroenterology.

[6] Rumilla K., Schowalter K.V., Lindor N.M., Thomas B.C., Mensink K.A., Gallinger S., Holter S., Newcomb P.A., Potter J.D., Jenkins M.A. (2011). Frequency of deletions of EPCAM (TACSTD1) in MSH2-associated Lynch syndrome cases. J. Mol. Diagn..

[7] Niessen R.C., Hofstra R.M.W., Westers H., Ligtenberg M.J.L., Kooi K., Jager P.O.J., de Groote M.L., Dijkhuizen T., Olderode-Berends M.J.W., Hollema H. (2009). Germline hypermethylation of MLH1 and EPCAM deletions are a frequent cause of Lynch syndrome. Genes Chromosomes Cancer.

[8] Ligtenberg M.J.L., Kuiper R.P., Chan T.L., Goossens M., Hebeda K.M., Voorendt M., Lee T.Y.H., Bodmer D., Hoenselaar E., Hendriks-Cornelissen S.J.B. (2009). Heritable somatic methylation and inactivation of MSH2 in families with Lynch syndrome due to deletion of the 3′ exons of TACSTD1. Nat. Genet..

[9] Nagasaka T., Rhees J., Kloor M., Gebert J., Naomoto Y., Boland C.R., Goel A. (2010). Somatic hypermethylation of MSH2 is a frequent event in Lynch Syndrome colorectal cancers. Cancer Res..

[10] Kloor M., Voigt A.Y., Schackert H.K., Schirmacher P., von Knebel Doeberitz M., Bläker H. (2011). Analysis of EPCAM protein expression in diagnostics of Lynch syndrome. J. Clin. Oncol..

[11] Huth C., Kloor M., Voigt A.Y., Bozukova G., Evers C., Gaspar H., Tariverdian M., Schirmacher P., von Knebel Doeberitz M., Bläker H. (2012). The molecular basis of EPCAM expression loss in Lynch syndrome-associated tumors. Mod. Pathol..

[12] Musulen E., Blanco I., Carrato C., Fernandez-Figueras M.T., Pineda M., Capella G., Ariza A. (2013). Usefulness of epithelial cell adhesion molecule expression in the algorithmic approach to Lynch syndrome identification. Hum. Pathol..

[13] Musulén E., Sanz C., Muñoz-Mármol A.M., Ariza A. (2014). Mismatch repair protein immunohistochemistry: A useful population screening strategy for Lynch syndrome. Hum. Pathol..

[14] Spaepen M., Neven E., Sagaert X., De Hertogh G., Beert E., Wimmer K., Matthijs G., Legius E., Brems H. (2013). EPCAM germline and somatic rearrangements in Lynch syndrome: Identification of a novel 3′EPCAM deletion. Genes Chromosomes Cancer.

[15] Kim J.H., Bae J.M., Song Y.S., Cho N.Y., Lee H.S., Kang G.H. (2016). Clinicopathologic, molecular, and prognostic implications of the loss of EPCAM expression in colorectal carcinoma. Oncotarget.

[16] Kempers M.J.E., Kuiper R.P., Ockeloen C.W., Chappuis P.O., Hutter P., Rahner N., Schackert H.K., Steinke V., Holinski-Feder E., Morak M. (2011). Risk of colorectal and endometrial cancers in EPCAM deletion-positive Lynch syndrome: A cohort study. Lancet Oncol..

[17] Perez-Cabornero L., Sanz M.I., Sampedro E.V., Aras E.L., Becares A.A., Pino C.M., Dominguez M.D. (2011). Frequency of rearrangements in lynch syndrome cases associated with MSH2: Characterization of a New deletion involving both EPCAM and the 5′ part of MSH2. Cancer Prev. Res..

[18] Ryan E., Sheahan K., Creavin B., Mohan H.M., Winter D.C. (2017). The current value of determining the mismatch repair status of colorectal cancer: A rationale for routine testing. Crit. Rev. Oncol. Hematol..

[19] Vangala D.B., Cauchin E., Balmaña J., Wyrwicz L., van Cutsem E., Güller U., Castells A., Carneiro F., Hammel P., Ducreux M. (2018). Screening and surveillance in hereditary gastrointestinal cancers: Recommendations from the European Society of Digestive Oncology (ESDO) expert discussion at the 20th European Society for Medical Oncology (ESMO)/World Congress on Gastrointestinal Cancer. Eur. J. Cancer.

[20] Schiffman M., Doorbar J., Wentzensen N., de Sanjosé S., Fakhry C., Monk B.J., Stanley M.A., Franceschi S. (2016). Carcinogenic human papillomavirus infection. Nat. Rev. Dis. Prim..

[21] Konstantinou E., Fotopoulou F., Drosos A., Dimakopoulou N., Zagoriti Z., Niarchos A., Makrynioti D., Kouretas D., Farsalinos K., Lagoumintzis G. (2018). Tobacco-specific nitrosamines: A literature review. Food Chem. Toxicol..

[22] Kahn R.M., Gordhandas S., Maddy B.P., Baltich Nelson B., Askin G., Christos P.J., Caputo T.A., Chapman-Davis E., Holcomb K., Frey M.K. (2019). Universal endometrial cancer tumor typing: How much has immunohistochemistry, microsatellite instability, and *MLH1* methylation improved the diagnosis of Lynch syndrome across the population?. Cancer.

[23] Ryan N.A.J., Glaire M.A., Blake D., Cabrera-Dandy M., Evans D.G., Crosbie E.J. (2019). The proportion of endometrial cancers associated with Lynch syndrome: A systematic review of the literature and meta-analysis. Genet. Med..

[24] Porkka N.K., Olkinuora A., Kuopio T., Ahtiainen M., Eldfors S., Almusa H., Mecklin J.-P., Peltomäki P. (2020). Does breast carcinoma belong to the Lynch syndrome tumor spectrum?—Somatic mutational profiles vs. ovarian and colorectal carcinomas. Oncotarget.

[25] Iino H., Simms L., Young J., Arnold J., Winship I.M., Webb S.I., Furlong K.L., Leggett B., Jass J.R. (2000). DNA microsatellite instability and mismatch repair protein loss in adenomas presenting in hereditary non-polyposis colorectal cancer. Gut.

[26] De Jong A.E., Morreau H., Van Puijenbroek M., Eilers P.H., Wijnen J., Nagengast F.M., Griffioen G., Cats A., Menko F.H., Kleibeuker J.H. (2004). The role of mismatch repair gene defects in the development of adenomas in patients with HNPCC. Gastroenterology.

[27] Sekine S., Mori T., Ogawa R., Tanaka M., Yoshida H., Taniguchi H., Nakajima T., Sugano K., Yoshida T., Kato M. (2017). Mismatch repair deficiency commonly precedes adenoma formation in Lynch Syndrome-Associated colorectal tumorigenesis. Mod. Pathol..

[28] Dabir P.D., Bruggeling C.E., van der Post R.S., Dutilh B.E., Hoogerbrugge N., Ligtenberg M.J.L., Boleij A., Nagtegaal I.D. (2020). Microsatellite instability screening in colorectal adenomas to detect Lynch syndrome patients? A systematic review and meta-analysis. Eur. J. Hum. Genet..

[29] Pino M.S., Mino-Kenudson M., Wildemore B.M., Ganguly A., Batten J., Sperduti I., Iafrate A.J., Chung D.C. (2009). Deficient DNA mismatch repair is common in lynch syndrome-associated colorectal adenomas. J. Mol. Diagn..

[30] van Lier M.G.F., Leenen C.H.M., Wagner A., Ramsoekh D., Dubbink H.J., van den Ouweland A.M.W., Westenend P.J., de Graaf E.J.R., Wolters L.M.M., Vrijland W.W. (2012). Yield of routine molecular analyses in colorectal cancer patients ≤70 years to detect underlying Lynch syndrome. J. Pathol..

[31] Walsh M.D., Buchanan D.D., Pearson S.-A., Clendenning M., Jenkins M.A., Win A.K., Walters R.J., Spring K.J., Nagler B., Pavluk E. (2012). Immunohistochemical testing of conventional adenomas for loss of expression of mismatch repair proteins in Lynch syndrome mutation carriers: A case series from the Australasian site of the colon cancer family registry. Mod. Pathol..

[32] Yurgelun M.B., Goel A., Hornick J.L., Sen A., Turgeon D.K., Ruffin M.T., Marcon N.E., Baron J.A., Bresalier R.S., Syngal S. (2012). Microsatellite instability and DNA mismatch repair protein deficiency in lynch syndrome colorectal polyps. Cancer Prev. Res..

[33] Halvarsson B., Lindblom A., Johansson L., Lagerstedt K., Nilbert M. (2005). Loss of mismatch repair protein immunostaining in colorectal adenomas from patients with hereditary nonpolyposis colorectal cancer. Mod. Pathol..

[34] Valo S., Kaur S., Ristimäki A., Renkonen-Sinisalo L., Järvinen H., Mecklin J.-P., Nyström M., Peltomäki P. (2015). DNA hypermethylation appears early and shows increased frequency with dysplasia in Lynch syndrome-associated colorectal adenomas and carcinomas. Clin. Epigenet..

[35] Basterra M., Gomez M., Mercado M.D.R., Irisarri R., Amorena E., Arrospide A., Montes M., Aisa G., Cambra K.I., Urman J. (2016). Prevalence of altered mismatch repair protein nuclear expression detected by immunohistochemistry on adenomas with high-grade dysplasia and features associated with this risk in a population-based study. Gastroenterol. Hepatol..

[36] Tanaka M., Nakajima T., Sugano K., Yoshida T., Taniguchi H., Kanemitsu Y., Nagino M., Sekine S. (2016). Mismatch repair deficiency in Lynch syndrome-associated colorectal adenomas is more prevalent in older patients. Histopathology.

[37] Meijer T.W.H., Hoogerbrugge N., Nagengast F.M., Ligtenberg M.J.L., van Krieken J.H.J.M. (2009). In Lynch syndrome adenomas, loss of mismatch repair proteins is related to an enhanced lymphocytic response. Histopathology.

[38] Sekine S., Ogawa R., Saito S., Ushiama M., Shida D., Nakajima T., Taniguchi H., Hiraoka N., Yoshida T., Sugano K. (2017). Cytoplasmic MSH2 immunoreactivity in a patient with Lynch syndrome with an EPCAM-MSH2 fusion. Histopathology.

[39] Sivagnanam M., Mueller J.L., Lee H., Chen Z., Nelson S.F., Turner D., Zlotkin S.H., Pencharz P.B., Ngan B.-Y., Libiger O. (2008). Identification of EpCAM as the gene for congenital tufting enteropathy. Gastroenterology.

[40] Pathak S.J., Mueller J.L., Okamoto K., Das B., Hertecant J., Greenhalgh L., Cole T., Pinsk V., Yerushalmi B., Gurkan O.E. (2019). *EPCAM* mutation update: Variants associated with congenital tufting enteropathy and Lynch syndrome. Hum. Mutat..

[41] Lynch H.T., Lynch J.F., Snyder C.L., Riegert-Johnson D. (2011). EPCAM deletions, Lynch syndrome, and cancer risk. Lancet Oncol..

[42] Grandval P., Baert-Desurmont S., Bonnet F., Bronner M., Buisine M.-P., Colas C., Noguchi T., North M.-O., Rey J.-M., Tinat J. (2012). Colon-specific phenotype in Lynch syndrome associated with EPCAM deletion. Clin. Genet..

[43] Schmelzer E., Reid L.M. (2008). EpCAM expression in normal, non-pathological tissues. Front. Biosci..

[44] Spizzo G., Fong D., Wurm M., Ensinger C., Obrist P., Hofer C., Mazzoleni G., Gastl G., Went P. (2011). EpCAM expression in primary tumour tissues and metastases: An immunohistochemical analysis. J. Clin. Pathol..

[45] Giuffrè G., Müller A., Brodegger T., Bocker-Edmonston T., Gebert J., Kloor M., Dietmaier W., Kullmann F., Büttner R., Tuccari G. (2005). Microsatellite analysis of hereditary nonpolyposis colorectal cancer-associated colorectal adenomas by laser-assisted microdissection: Correlation with mismatch repair protein expression provides new insights in early steps of tumorigenesis. J. Mol. Diagn..

[46] Shia J., Stadler Z.K., Weiser M.R., Vakiani E., Mendelsohn R., Markowitz A.J., Shike M., Boland C.R., Klimstra D.S. (2015). Mismatch repair deficient-crypts in non-neoplastic colonic mucosa in Lynch syndrome: Insights from an illustrative case. Fam. Cancer.

[47] Pai R.K., Dudley B., Karloski E., Brand R.E., O’Callaghan N., Rosty C., Buchanan D.D., Jenkins M.A., Thibodeau S.N., French A.J. (2018). DNA mismatch repair protein deficient non-neoplastic colonic crypts: A novel indicator of Lynch syndrome. Mod. Pathol..

[48] Susswein L.R., Marshall M.L., Nusbaum R., Postula K.J.V., Weissman S.M., Yackowski L., Vaccari E.M., Bissonnette J., Booker J.K., Cremona M.L. (2016). Pathogenic and likely pathogenic variant prevalence among the first 10,000 patients referred for next-generation cancer panel testing. Genet. Med..

[49] Thompson B.A., Spurdle A.B., Plazzer J.P., Greenblatt M.S., Akagi K., Al-Mulla F., Bapat B., Bernstein I., Capellá G., den Dunnen J.T. (2014). Application of a 5-tiered scheme for standardized classification of 2360 unique mismatch repair gene variants in the InSiGHT locus-specific database. Nat. Genet..

