# Supplementary material for: Complete Loss of EPCAM Immunoexpression Identifies EPCAM Deletion Carriers in MSH2-Negative Colorectal Neoplasia
Source: Cancers (Basel). 2020 Sep 29;12(10):2803. doi: 10.3390/cancers12102803 (PMC7599495; doi:10.3390/cancers12102803)
Supplement: Supplementary file 1 [file cancers-12-02803-s001.pdf]

Supplementary Materials:

Complete Loss of EPCAM Immunoexpression Identifies *EPCAM* Deletion Carriers in MSH2-Negative Colorectal Neoplasia

Table S1. Clinicopathological features and molecular alterations of all cases.

Case	Patient	Family	Gender	Age# (years)	Lesion	Histology	Location	MSH2 Expression	EPCAM Expression	EPCAM Evaluation	Mutated Gene	Chromosomical Alteration	Significant
1α	1	1	M	31	MA	Mucinous	Right colon	-	+		*EPCAM*	Deletion of exons 8-9	Pathogenic
2	1			34	CP	HP	Right colon	+	+				
3α	2	1	M	62	MA	Mucinous	Right colon	-	-	I	*EPCAM*	Deletion of exons 8-9	Pathogenic
4	2			63	CP	HP	Right colon	+	+				
5	2			63	CP	SSA	Right colon	-	+				
6	2			64	CP	SSA	Rectum	-	-*	I			
7	2			65	CP	TSA	Rectum	-	-*	I			
8	2			66	CP	SSA	Rectum	-	+				
9	2			67	CP	SSA	Rectum	-	-*	I			
10α	3	1	M	43	MA	Medullary	Right colon	-	+		*EPCAM*	Deletion of exons 8-9	Pathogenic
11α	3			43	MA	Mucinous	Left colon	-	+				
12	3			43	CP	SSA	Left colon	-	-	I			
13	3			43	CP	TVA	Left colon	-	+				
14α	4	1	F	43	MA	ADC NOS	Right colon	-	-	I	*EPCAM*	Deletion of exons 8-9	Pathogenic
15	4			43	CP	TSA	Right colon	-	+				
16	4			45	CP	SSA	Right colon	-	+				
17α	5	1	M	45	MA	ADC NOS	Right colon	-	+		*EPCAM*	Deletion of exons 8-9	Pathogenic
18α	5			45	MA	Mucinous	Right colon	-	+				
19α	5			45	MA	Mucinous	Left colon	-	-	I			
20	5			45	CP	TA	Right colon	-	+				
21	5			45	CP	TA	Right colon	-	+				
22	5			54	CP	TA	Rectum	-	-*	I			
23α	6	1	M	44	MA	Mucinous	Right colon	-	-	I	*EPCAM*	Deletion of exons 8-9	Pathogenic
24α	6			44	MA	Mucinous	Right colon	-	-	I			
25α	6			44	MA	Mucinous	Left colon	-	+				
26	6			45	CP	SSA	Right colon	-	+				

27	7	2	F	61	MA	ADC NOS	Left colon	-	+		*EPCAM*	Deletion of exons 8-9	Pathogenic
28	8	3	F	52	MA	ADC NOS	Duodenum	-	+		*EPCAM*	Deletion of exons 8-9	Pathogenic
29	9	4	F	43	MA	ADC NOS	Colon	-	+		*EPCAM*	Deletion of exons 8-9	Pathogenic
30	9			63	MA	ADC NOS	Colon	-	+				
31	10	4	F	31	MA	ADC NOS	Colon	-	-	I	*EPCAM*	Deletion of exons 8-9	Pathogenic
32	10			31	P	HSIL	Cervix	+	-	NI			
33	10			31	P	HSIL	Cervix	+	-	NI			
34	11	4	M	33	MA	ADC NOS	Colon	-	-	I	*EPCAM*	Deletion of exons 8-9	Pathogenic
35	11			33	MA	ADC NOS	Colon	-	-	I			
36	11			33	MA	ADC NOS	Colon	-	-	I			
37	12	5	F	56	MA	ADC NFS	Endometrium	NA	NA		*EPCAM*	Deletion of exons 8-9	Pathogenic
38	12			75	MA	Mucinous and medullary	Right colon	-/C	-	I			
39	12			80	MA	ADC NFS	Colon	NA	NA				
40	13	5	M	54	MA	ADC PD	Stomach	-/C	+		*EPCAM*	Deletion of exons 8-9	Pathogenic
41	14	6	F	39	MA	Clear cell C	Ovary	-	+		*EPCAM-MSH2*	Deletion of exons 8-9 of *EPCAM* and exons 1-2 of *MSH2*	Pathogenic
42	14			40	CP	TA	Right colon	-	-	I			
43	14			42	CP	TA	Right colon	-	+				
44	14			42	CP	TA	Colon	-	-*	I			
45	15	6	F	22	MA	Hodgkin Lymphoma	Unknown	NA	NA		*EPCAM-MSH2*	Deletion of exons 8-9 of *EPCAM* and exons 1-2 of *MSH2*	Pathogenic
46	15			38	MA	Endometrioid C	Endometrium	-	+				Pathogenic
47	16		M	40	MA	Squamous Cell C	Skin	-	-	NI	*MSH2*	c.689_691delinsTT; p.(Ala230Valfs*16)	Pathogenic
48	16			41	MA	Urothelial C	Urinary bladder	-	+				
49	16			42	CP	SSA	Rectum	-	+				
50	16			47	MA	Squamous Cell C	Skin	-	-	NI			
51	16			48	MA	Urothelial C	Urinary bladder	-	+				
52	17		F	57	CP	TA	Right colon	-	+		*MSH2*	Duplication of exon 11; p.Gly587Alafs*3	Pathogenic
53	17			57	CP	SSA	Left colon	+	+				
54	18		F	55	CP	TA	Right colon	+	+		*MSH2*	c.897T>A; p.(Tyr299*)	Pathogenic
55	18			55	CP	TA	Left colon	+	+				
56	18			55	CP	TA	Right colon	+	+				
57	18			55	B	Sebaceoma	Skin	-	-	NI			
58	18			55	B	SA	Skin	-	-	NI			

59	18		55	B	SA	Skin	-	-	NI			
60	18		56	CP	TA	Right colon	+	+				
61	18		56	CP	SSA	Right colon	+	+				
62	18		58	CP	TA	Right colon	+	+				
63	18		58	CP	SSA	Right colon	+	+				
64	18		59	CP	SSA	Rectum	+	+				
65	19	F	62	CP	TA	Left colon	-	+		*MSH2*	c.689_691delinsTT; p.(Ala230Valfs*16)	Pathogenic
66	19		62	CP	TA	Left colon	+	+				
67	19		63	MA	Medullary	Colon	-	+				
68	19		63	CP	SSA	Appendix	+	+				
69	19		64	MA	Endometrioid C	Endometrium	-	+				
70	20	M		MA	Neuroendocrine	Colon	-	+		*MSH2*	c.689_691delinsTT; p.(Ala230Valfs*16)	Pathogenic
71	21	M	46	MA	ADC NOS	Colon	-	+		*MSH2*	c.211G>C; p.[?,Tyr66Serfs*10]	Pathogenic
72	22	F	81	CP	TSA	Right colon	-	+		*MSH2*	691delinsTT; p.(Ala230Valfs*16)	Pathogenic
73	22		81	CP	TSA	Rectum	-	+				
74	22		82	MA	ADC NOS	Colon	-	+				
75	22		82	CP	TA	Right colon	-	+				
76	22		82	CP	SSA	Right colon	+	+				
77	23	F	44	MA	Medullary	Colon	-	+		*MSH2*	c.518T>G; p(Leu173Arg)	Pathogenic
78	23		45	CP	TA	Rectum	-	+				
79	24	M	31	MA	Mucinous	Right colon	-	+		*MSH2*	c.2415_2421dup; p.(Glu808Hisfs*3)	Pathogenic
80	25	M	47	MA	ADC NOS	Left colon	-	+		*MSH2*	c.1226_1227del; p.(Gln409Argfs*7)	Pathogenic
81	26	M	61	CP	SSA	Colon	-	+		*MSH2*	c.536dup; p.(Asp180*)	Pathogenic
82	26		61	MA	ADC NOS	Right colon	-	+				
83	26		62	MA	ADC NOS	Right colon	-	+				
84	26		67	CP	SSA	Colon	+	+				
85	26		71	MA	ADC NOS	Colon	-	+				
86	26		75	CP	TA	Right colon	-	+				
87	26		77	CP	TA	Right colon	-	+				
88	26		81	MA	ADC NOS	Left colon	-	+				
89	26		81	CP	TA	Rectum	-	+		*MSH2*		
90	27	F	57	MA	ADC NOS	Rectum	-	+		*MSH2*	c. 223dup; p.(Leu75Profs*7)	Pathogenic

91	28	M	44	MA	ADC NOS	Rectum	-	+	*MSH2*	c.536dup; p.(Asp180*)	Pathogenic
92	29	M	42	MA	Medullary C	Rectum	-	+	*MSH2*	c. 223dup; p.(Leu75Profs*7)	Pathogenic
93	30	F	49	MA	Endometrioid C	Endometrium	-	+	*MSH2*	c.732delG; p.(Leu244Phefs*2)	Pathogenic
94	30		49	P	CH	Endometrium	-	+			
95	31	F	45	MA	Mucinous	Right colon	-	+	*MSH2*	c.731_734del; p.(Leu244*)	Pathogenic
96	32	F	36	MA	ADC NOS	Right colon	-	+	*MSH2*	c.2593dup; p.(Ile865Asnfs*17)	Pathogenic
97	33	F	46	MA	Serous C	Endometrium	-	+	*MSH2*	c.1216C>T; p.(Arg406*)	Pathogenic
98	34	F	48	MA	Endometrioid C	Endometrium	-	+	*MSH2*	c.942+3A>T; p.Val265_Gln314del	Pathogenic
99	34		48	P	SH	Endometrium	-	+			
100	35	M	65	MA	Urothelial C	Urinary bladder	+	+	*MSH2*	NFS	Pathogenic
101	36	F	43	CP	TA	Right colon	-	+	*MSH2*	c.536_537insC; p.Asp180X	Pathogenic
102	36		43	CP	TA	Right colon	-	+			
103	36		43	CP	HP	Right colon	+	+			
104	36		43	MA	ADC NOS	Colon	-	+			
105	36		44	P	SH	Endometrium	-	+			
106	36		46	CP	TA	Right colon	+	+			
107	36		48	CP	SSA	Rectum	+	+			
108	37	F	37	MA	ADC NOS	Endometrium	-	+	*MSH2*	c.536_537insC; p.Asp180X	Pathogenic
109	38	F	39	CP	TA	Colon	-	+	*MSH2*	Deletion of exons 9-10	Pathogenic
110	38		43	CP	HP	Left colon	+	+			
111	39	F	24	CP	TA	Left colon	-	+	*MSH2*	c.732delG; p.(Leu244Phefs*2)	Pathogenic
112	39		24	MA	ADC NOS	Left colon	-	+			
113	39		24	CP	SSA	Left colon	-	+			
114	40	F	63	CP	TA	Left colon	-	+	*MSH2*	c.2222_2223delAA; p.(Lys741Argfs*8)	Pathogenic
115	40		63	MA	Mucinous	Duodenum	-	+			
116	40		64	MA	ADC NOS	Colon	-	+			
117	41	M	53	MA	ADC NOS	Rectum	-	+	*MSH2*	Deletion of exons 9-10	Pathogenic
118	41		53	CP	SSA	Rectum	-	+			
119	41		53	CP	SSA	Left colon	-	+			
120	42	M	32	MA	ADC NOS	Right colon	-	+	*MSH2*	c.1662-2A>G; p.?	Pathogenic

121	43	F	36	MA	Endometrioid C	Endometrium	+	+		*MSH2*	Deletion of exon 8; p.Lys427Glyfs*4	Pathogenic
122	44	F	56	MA	Endometrioid C	Endometrium	-	+		*MSH2*	c.2164_2192del; p.(Val722Asnfs*18)	Pathogenic
123	44		59	CP	SSA	Right colon	+	+				
124	45	M	36	MA	Mucinous	Right colon	-	+		*MSH2*	c.2074G>C; p.(Gly692Arg)	Pathogenic
125	46	F	53	MA	Ductal C	Breast	+	-	NI	*MSH2*	c.2635-3C>T;2635-5T> C]; p.?	Pathogenic
126	46		53	P	Ductal *in situ* C	Breast	+	-	NI			
127	47	F	39	MA	Clear cell C	Unknown	-	+		*MSH2*	c.[2635-3C>T;2635-5T> C]; p.?	Pathogenic
128	48	F	48	P	CH	Endometrium	-	+		*MSH2*	c.1705_1706delGA; p.(Glu569Ilefs*2)	Pathogenic
129	48		48	MA	Endometrioid C	Endometrium	-	+				
130	49	F	42	MA	Endometrioid C	Endometrium	-	+		*MSH2*	c.367-6_370dup; p.(Ser124Tyrfs*12)	Pathogenic
131	49		42	P	CH	Endometrium	-	+				
132	50	M	58	MA	Squamous Cell C	Skin	-	-	NI	*MSH2*	c.1705_1706delGA; p.(Glu569Ilefs*2)	Pathogenic
133	51	F	50	MA	Endometrioid C	Endometrium	-	+		*MSH2*	c.1705_1706delGA; p.(Glu569Ilefs*2)	Pathogenic
134	51		50	MA	Clear cell C	Endometrium	-	+				
135	52	F	45	MA	Medullary	Colon	-	+		*MSH2*	c.536dup; p.(Asp180*)	Pathogenic
136	52		51	MA	Squamous Cell C	Skin	-	-	NI			
137	53	F	37	MA	Medullary	Right colon	-	+		*MSH2*	c.970C>T; p.(Gln324*)	Pathogenic
138	54	F	42	MA	Undifferentiated C	Ovary	-	+		*MSH2*	c.1276G>A; p.Ile411_Gly426del16	Pathogenic
139	54		42	MA	ADC NOS	Right colon	-	+				
140	55	M	48	MA	ADC NOS	Right colon	-	+		*MSH2*	Deletion of exons 9-10	Pathogenic
141	55		48	CP	TA	Right colon	-	+				
142	55		48	CP	TA	Right colon	-	+				
143	55		48	CP	TA	Right colon	-	+				
144	55		48	MA	ADC NOS	Right colon	-	+				
145	55		57	MA	ADC NOS	Prostate	-	+				
146	56	M	41	MA	Squamous Cell C	Skin	-	-	NI	*MSH2*	Deletion of exons 9-10	Pathogenic
147	57	M	56	MA	Squamous Cell C	Lip	-	-	NI	*MSH2*	c.536dup; p.(Asp180*)	Pathogenic
148	58	F	**28**	MA	ADC NOS	Endometrium	-	+		*MSH2*	c.1276G>A; p.Ile411_Gly426del16	Pathogenic

149	59	M	42	MA	Mucinous	Right colon	-	+		*MSH2*	c.942+3A>T; p.Val265_Gln314del	Pathogenic
150	59		42	MA	Mucinous	Right colon	-	+				
151	60	F	50	MA	Endometrioid C	Endometrium	-	+		*MSH2*	Deletion of exon 8; p.Lys427Glyfs*4	Pathogenic
152	60		50	P	CH	Endometrium	-	+				
153	60		54	MA	ADC NOS	Right colon	-	+				
154	61	F	53	MA	Undifferentiated C	Endometrium	+	+		*MSH2*	c.1980_1981delTA; p.(Asp660Glufs*15)	Pathogenic
155	62	F	33	MA	Endometrioid C	Endometrium	-	+		*MSH2*	c.1035G>A; c.1035G>A	Pathogenic
156	63	F	61	MA	Urothelial C	Renal pelvis	-	+		*MSH2*	c.1345_1348delAAG;p.(Lys449Phefs*4)	Pathogenic
157	63		61	MA	ADC NOS	Pancreas	-	+				
158	64	M	57	MA	ADC NOS	Colon	-	+		*MSH2*	c.[2635-3C>T;2635-5T>C]; p.?	Pathogenic
159	64		57	CP	TA	Right colon	+	+				
160	64		57	CP	TA	Right colon	-	+				
161	64		57	CP	TA	Right colon	+	+				
162	64		65	MA	Undifferentiated C	Adrenal gland	-	-	NI			
163	65	M	50	MA	Mucinous	Colon	-	+		*MSH2*	c.1076+1G>A; p.Gly315Ilefs*29	Pathogenic
164	65		50	MA	Urothelial C	Urinary bladder	+	+				
165	65		50	MA	Urothelial C	Ureter	-	+				
166	65		52	B	Sebaceoma	Skin	-	-	NI			
167	65		52	MA	Basal cell C	Skin	-	-	NI			
168	65		53	CP	TA	Rectum	+	+				
169	65		53	B	Sebaceoma	Skin	-	-	NI			
170	65		54	MA	Urothelial C	Urinary bladder	-	+				
171	65		54	B	SA	Skin	-	-	NI			
172	65		54	B	SA	Skin	-	-	NI			
173	66	M	40	MA	ADC NOS	Rectum	-	+		*MSH2*	c.528_529delTG; p.(Cys176*)	Pathogenic
174	67	M	49	MA	ADC NOS	Rectum	-	+		*MSH2*	c.[2635-3C>T;2635-5T>C]; p.?	Pathogenic
175	67		53	B	SA	Skin	-	-	NI			
176	68	F	35	MA	ADC NOS	Colon	-	+		*MSH2*	c.2069A>G; p.(Gln690Arg)	Pathogenic
177	68		43	CP	SSA	Colon	-	+				
178	68		45	CP	SSA	Colon	-	+				

179	68			52	CP	TVA	Right colon	-	+				
180	68			53	MA	ADC NOS	Colon	-	+				
181	68			54	CP	TA	Colon	-	+				
182	68			54	CP	SSA	Colon	-	+				
183	68			58	MA	ADC NOS	Colon	-	+				
184	68			61	CP	TA	Colon	-	+				
185	68			62	MA	Medullary	Right colon	-	+				
186	69		F	54	CP	SSA	Right colon	+	+		*MSH2*	NFS	VUS
187	69			55	CP	SSA	Left colon	+	+				
188	69			57	CP	TA	Right colon	-	+				
189	70		F	47	MA	Endometrioid C	Endometrium	-	+		Unknown	No mutation found	
190	71		F	33	MA	Mucinous	Left colon	-	+		Unknown	No mutation found	

Abbreviations: α, cases included in previous series (refs.12,13); #, age at diagnosis; F, female; M, male; B, benign; P, precursor; CP, colorectal polyp; MA, malignant; ADC NOS, adenocarcinoma not otherwise specified; ADC PD, adenocarcinoma poorly differentiated; C, carcinoma; CH, complex hyperplasia; SH, simplex hyperplasia; HP, hyperplastic polyp; TA, tubular adenoma; SSL: sessile serrated lesion; TSA, traditional serrated adenoma; +, positive; -, negative; -*, focal negative; C, cytoplasmic; I, informative; NI, noninformative; NFS, not further specified; VUS, variant of unknown significance; NA, not available.

Table S2. Clinicopathological features and molecular alterations of colorectal polyps.

Case	Patient	Family	Gender	Age	Histology	Location	Size (mm)	Dysplasia	MSH2 Expression	EPCAM Expression	Mutated Gene
1	1	Family 1	M	34	HP	Right colon	1	WD	+	+	*EPCAM*
2	2	Family 1	M	63	HP	Right colon	5	WD	+	+	*EPCAM*
3	2	Family 1		63	SSL	Right colon	4	HGD	-	+	
4	2	Family 1		64	SSL	Rectum	5	HGD	-	-*	
5	2	Family 1		65	TSA	Rectum	8	HGD	-	-*	
6	2	Family 1		66	SSL	Rectum	14	HGD	-	+	
7	2	Family 1		67	SSL	Rectum	10	HGD	-	-*	
8	3	Family 1	M	43	SSL	Left colon	7	HGD	-	-	*EPCAM*
9	3	Family 1		43	TVA	Left colon	6	HGD	-	+	
10	4	Family 1	F	43	TSA	Right colon	8	HGD	-	+	*EPCAM*
11	4	Family 1		45	SSL	Right colon	10	HGD	-	+	
12	5	Family 1	M	45	TA	Right colon	4	HGD	-	+	*EPCAM*
13	5	Family 1		45	TA	Right colon	4	HGD	-	+	
14	5	Family 1		54	TA	Rectum	5	HGD	-	-*	
15	6	Family 1	M	45	SSL	Right colon	2	LGD	-	+	*EPCAM*
16	14	Family 6	F	40	TA	Right colon	15	HGD	-	-	*EPCAM-MSH2*
17	14	Family 6		42	TA	Right colon	12	LGD	-	+	

18	14	Family 6		42	TA	Colon	7	LGD	-	-*	
19	16		M	42	SSL	Rectum	6	WD	-	+	*MSH2*
20	17		F	57	TA	Right colon	11	LGD	-	+	*MSH2*
21	17			57	SSL	Left colon	10	LGD	+	+	
22	18		F	55	TA	Right colon	1	HGD	+	+	*MSH2*
23	18			55	TA	Left colon	5	LGD	+	+	
24	18			55	TA	Right colon	10	HGD	+	+	
25	18			56	TA	Right colon	3	HGD	+	+	
26	18			56	SSL	Right colon	4	HGD	+	+	
27	18			58	TA	Right colon	2	LGD	+	+	
28	18			58	SSL	Right colon	10	HGD	+	+	
29	18			59	SSL	Rectum	3	WD	+	+	
30	19		F	62	TA	Left colon	1	LGD	-	+	*MSH2*
31	19			62	TA	Left colon	2	LGD	+	+	
32	19			63	SSL	Appendix	2	WD	+	+	
33	22		F	81	TSA	Right colon	4	HGD	-	+	*MSH2*
34	22				TSA	Rectum	12	HGD	-	+	
35	22				TA	Right colon	5	HGD	-	+	
36	22				SSL	Right colon	14	HGD	+	+	
37	23		F	45	TA	Rectum	8	HGD	-	+	*MSH2*
38	26		M	61	SSL	Colon	6	HGD	-	+	*MSH2*
39	26			67	SSL	Colon	8	HGD	+	+	
40	26			75	TA	Right colon	7	LGD	-	+	
41	26			77	TA	Right colon	4	HGD	-	+	
42	26			81	TA	Rectum	10	HGD	-	+	
43	36		F	43	TA	Right colon	4	HGD	-	+	*MSH2*
44	36			43	TA	Right colon	8	HGD	-	+	
45	36			43	HP	Right colon	8	WD	+	+	
46	36			46	TA	Right colon	10	LGD	+	+	
47	36			48	SSL	Rectum	3	WD	+	+	
48	38		F	39	TA	Colon	9	LGD	-	+	*MSH2*
49	38			43	HP	Left colon	3	WD	+	+	
50	39		F	24	TA	Left colon	22	LGD	-	+	*MSH2*
51	40		F	63	TA	Left colon	10	HGD	-	+	*MSH2*
52	41		M	53	SSL	Rectum	5	HGD	-	+	*MSH2*
53	44		F	59	SSL	Right colon	22	LGD	+	+	*MSH2*
54	39		F	24	SSL	Left colon	20	LGD	-	+	*MSH2*
55	41		M	53	SSL	Left colon	20	HGD	-	+	*MSH2*
56	55		M	48	TA	Right colon	3	LGD	-	+	*MSH2*

57	55		48	TA	Right colon	15	LGD	-	+	
58	55		48	TA	Right colon	10	LGD	-	+	
59	64	M	57	TA	Right colon	1	LGD	+	+	*MSH2*
60	64		57	TA	Right colon	9	LGD	-	+	
61	64		57	TA	Right colon	9	LGD	+	+	
62	65	M	53	TA	Rectum	2	LGD	+	+	*MSH2*
63	68	F	43	SSL	Colon	8	LGD	-	+	*MSH2*
64	68		45	SSL	Colon	12	LGD	-	+	
65	68		52	TVA	Right colon	15	HGD	-	+	
66	68		54	TA	Colon	8	LGD	-	+	
67	68		54	SSL	Colon	7	LGD	-	+	
68	68		61	TA	Colon	5	LGD	-	+	
69	69	F	54	SSL	Right colon	2	WD	+	+	*MSH2*
70	69		55	SSL	Left colon	3	WD	+	+	
71	69		57	TA	Right colon	6	LGD	-	+	

Abbreviations: F, female; M, male; TA, tubular adenoma; TVA, tubulovillous adenoma; HP, hyperplastic polyp; SSL, sessile serrated lesion; TSA, traditional serrated adenoma; WD, without dysplasia; LGD, low grade dysplasia; HGD, high grade dysplasia; +, positive; -, negative; -*, focal negative.
